# Draft Genome Sequence of Aeromonas popoffii ID682, Isolated from a Natural Water Source in Idaho

**DOI:** 10.1128/MRA.00445-21

**Published:** 2021-07-22

**Authors:** Todd Testerman, Stephen R. Reichley, Stacy King, Joerg Graf

**Affiliations:** a Department of Molecular and Cell Biology, University of Connecticut, Storrs, Connecticut, USA; b Riverence, Buhl, Idaho, USA; Montana State University

## Abstract

A draft genome sequence of Aeromonas popoffii ID682, isolated from a natural water source in Idaho, is presented here. *A. popoffii* is a relatively understudied species within a diverse, expanding freshwater genus of bacteria. Here, we describe only the second genome published for this species to date.

## ANNOUNCEMENT

*Aeromonas* is a genus of Gram-negative bacteria found ubiquitously in aquatic environments (1). Many *Aeromonas* species have been shown to be opportunistic pathogens of a number of animal species (1–3) and present a treatment challenge due to developed antimicrobial resistance profiles (4, 5). Simultaneously, *Aeromonas* species have also been shown to be important beneficial symbionts of a number of organisms (6, 7). The species Aeromonas popoffii was first isolated from drinking water (8) and has been identified as an opportunistic human pathogen as well (9, 10). Here, we present a draft genome sequence of the novel strain Aeromonas popoffii ID682, isolated from a natural water source in Idaho.

*A. popoffii* ID682 was isolated from a surface swab sample of a submerged stone within a spring-fed pond draining into the Snake River using R2A agar (11) and incubated at 19°C. A single colony was streaked for isolation two consecutive times onto R2A agar to obtain a pure culture before being stocked at −80°C in a glycerol solution. Genomic DNA was extracted using the MasterPure DNA and RNA purification kit (Lucigen, Middleton, WI). DNA for all steps was quantified using the Qubit 1X double-stranded DNA (dsDNA) high-sensitivity (HS) assay kit (Invitrogen, Waltham, MA). A genome library was constructed using a DNA prep kit (Illumina, San Diego, CA) followed by 2 × 250-bp paired-end sequencing on a MiSeq device using a MiSeq reagent kit V2 (500 cycles) (Illumina, San Diego, CA). Following sequencing, raw reads were demultiplexed on BaseSpace and then downloaded for further processing.

CLC Genomics Workbench version 12.0.2 (Qiagen, Hilden, Germany) was used for read trimming (maximum ambiguities = 2, minimum accuracy = 95%, minimum read length = 60 bp) and *de novo* genome assembly (minimum contig length = 200, word size = 21, bubble size = 241) with default parameters. The draft genome sequence of *A. popoffii* ID682 was 4.68 Mb in size and was assembled into 128 contigs with an *N*_50_ value of 112 kbp. The G+C content was 58.6%, and the average genome coverage was 85× (Table 1).

**TABLE 1:** Genome summary and database accession information

Characteristic	Data for Aeromonas popoffii ID682
No. of raw reads	1,670,148
No. of reads in assembly	1,648,648
Genome size (Mbp)	4.68
Genome coverage (×)	85
No. of contigs	128
*N*_50_ (bp)	112,388
G+C content (%)	58.6
No. of coding sequences	4,472
No. of protein-coding sequences	4,180
No. of tRNAs	90
No. of rRNAs	8
Closest neighbor, whole genome (% dDDH)	Aeromonas popoffii CIP 105493 (84.3)
BioProject accession no.	PRJNA720925
BioSample accession no.	SAMN18685274
SRA accession no.	SRR14245844
GenBank accession no.	JAGRZL000000000

The genome was annotated using the NCBI Prokaryotic Genome Annotation Pipeline version 5.1 (12). A total of 4,648 coding sequences were identified. Secondary metabolite gene detection was performed using antiSMASH version 5.1.2 with relaxed detection strictness (13–17). Aryl polyene, bacteriocin, homoserine lactone, and nonribosomal peptide synthetase gene clusters were identified. Species-level identification was performed with digital DNA-DNA hybridization (dDDH) using TYGS version 267 (18). The closest genome to the subject strain was Aeromonas popoffii CIP 105493, with a dDDH of 84.3% when using the recommended d_4_ formula.

### Data availability.

The genome of *A. popoffii* ID682 has been deposited in DDBJ/ENA/GenBank under accession number JAGRZL000000000. The version described in this paper is the first version, JAGRZL010000000. The BioProject, BioSample, SRA, and GenBank accession numbers are provided in Table 1.

## References

[1] Janda JM, Abbott SL. 2010. The genus Aeromonas: taxonomy, pathogenicity, and infection. Clin Microbiol Rev 23:35–73. doi:10.1128/CMR.00039-09. 20065325 PMC2806660

[2] Igbinosa IH, Igumbor EU, Aghdasi F, Tom M, Okoh AI. 2012. Emerging Aeromonas species infections and their significance in public health. Sci World J 2012:625023. doi:10.1100/2012/625023. PMC3373137 22701365

[3] Menanteau-Ledouble S, Kumar G, Saleh M, El-Matbouli M. 2016. Aeromonas salmonicida: updates on an old acquaintance. Dis Aquat Org 120:49–68. doi:10.3354/dao03006. 27304870

[4] Aravena-Román M, Inglis TJJ, Henderson B, Riley TV, Chang BJ. 2012. Antimicrobial susceptibilities of Aeromonas strains isolated from clinical and environmental sources to 26 antimicrobial agents. Antimicrob Agents Chemother 56:1110–1112. doi:10.1128/AAC.05387-11. 22123695 PMC3264277

[5] Beka L, Fullmer MS, Colston SM, Nelson MC, Talagrand-Reboul E, Walker P, Ford B, Whitaker IS, Lamy B, Gogarten JP, Graf J. 2018. Low-level antimicrobials in the medicinal leech select for resistant pathogens that spread to patients. mBio 9:e01328-18. doi:10.1128/mBio.01328-18. 30042201 PMC6058295

[6] Graf J. 1999. Symbiosis of Aeromonas veronii biovar sobria and Hirudo medicinalis, the medicinal leech: a novel model for digestive tract associations. Infect Immun 67:1–7. doi:10.1128/IAI.67.1.1-7.1999. 9864188 PMC96269

[7] Sugita H, Tanaka K, Yoshinami M, Deguchi Y. 1995. Distribution of Aeromonas species in the intestinal tracts of river fish. Appl Environ Microbiol 61:4128–4130. doi:10.1128/aem.61.11.4128-4130.1995. 16535171 PMC1388607

[8] Huys G, Kämpfer P, Altwegg M, Kersters I, Lamb A, Coopman R, Lüthy-Hottenstein J, Vancanneyt M, Janssen P, Kersters K. 1997. Aeromonas popoffii sp. nov., a mesophilic bacterium isolated from drinking water production plants and reservoirs. Int J Syst Bacteriol 47:1165–1171. doi:10.1099/00207713-47-4-1165. 9336924

[9] Hua HT, Bollet C, Tercian S, Drancourt M, Raoult D. 2004. Aeromonas popoffii urinary tract infection. J Clin Microbiol 42:5427–5428. doi:10.1128/JCM.42.11.5427-5428.2004. 15528763 PMC525237

[10] Soler L, Figueras MJ, Chacón MR, Vila J, Marco F, Martinez-Murcia AJ, Guarro J. 2002. Potential virulence and antimicrobial susceptibility of Aeromonas popoffii recovered from freshwater and seawater. FEMS Immunol Med Microbiol 32:243–247. doi:10.1111/j.1574-695X.2002.tb00560.x. 11934570

[11] Reasoner DJ, Geldreich EE. 1985. A new medium for the enumeration and subculture of bacteria from potable water. Appl Environ Microbiol 49:1–7. doi:10.1128/aem.49.1.1-7.1985. 3883894 PMC238333

[12] Tatusova T, Dicuccio M, Badretdin A, Chetvernin V, Nawrocki EP, Zaslavsky L, Lomsadze A, Pruitt KD, Borodovsky M, Ostell J. 2016. NCBI Prokaryotic Genome Annotation Pipeline. Nucleic Acids Res 44:6614–6624. doi:10.1093/nar/gkw569. 27342282 PMC5001611

[13] Medema MH, Blin K, Cimermancic P, De Jager V, Zakrzewski P, Fischbach MA, Weber T, Takano E, Breitling R. 2011. AntiSMASH: rapid identification, annotation and analysis of secondary metabolite biosynthesis gene clusters in bacterial and fungal genome sequences. Nucleic Acids Res 39:W339–W346. doi:10.1093/nar/gkr466. 21672958 PMC3125804

[14] Blin K, Medema MH, Kazempour D, Fischbach MA, Breitling R, Takano E, Weber T. 2013. antiSMASH 2.0: a versatile platform for genome mining of secondary metabolite producers. Nucleic Acids Res 41:W204–W212. doi:10.1093/nar/gkt449. 23737449 PMC3692088

[15] Weber T, Blin K, Duddela S, Krug D, Kim HU, Bruccoleri R, Lee SY, Fischbach MA, Müller R, Wohlleben W, Breitling R, Takano E, Medema MH. 2015. AntiSMASH 3.0: a comprehensive resource for the genome mining of biosynthetic gene clusters. Nucleic Acids Res 43:W237–W243. doi:10.1093/nar/gkv437. 25948579 PMC4489286

[16] Blin K, Wolf T, Chevrette MG, Lu X, Schwalen CJ, Kautsar SA, Suarez Duran HG, De Los Santos ELC, Kim HU, Nave M, Dickschat JS, Mitchell DA, Shelest E, Breitling R, Takano E, Lee SY, Weber T, Medema MH. 2017. AntiSMASH 4.0: improvements in chemistry prediction and gene cluster boundary identification. Nucleic Acids Res 45:W36–W41. doi:10.1093/nar/gkx319. 28460038 PMC5570095

[17] Blin K, Shaw S, Steinke K, Villebro R, Ziemert N, Lee SY, Medema MH, Weber T. 2019. AntiSMASH 5.0: updates to the secondary metabolite genome mining pipeline. Nucleic Acids Res 47:W81–W87. doi:10.1093/nar/gkz310. 31032519 PMC6602434

[18] Meier-Kolthoff JP, Göker M. 2019. TYGS is an automated high-throughput platform for state-of-the-art genome-based taxonomy. Nat Commun 10:2182. doi:10.1038/s41467-019-10210-3. 31097708 PMC6522516

